# Supplementary figures and images for: From hidden hearing loss to supranormal auditory processing by neurotrophin 3-mediated modulation of inner hair cell synapse density
Source: PLoS Biol. 2024 Jun 27;22(6):e3002665. doi: 10.1371/journal.pbio.3002665 (PMC11210788; doi:10.1371/journal.pbio.3002665)

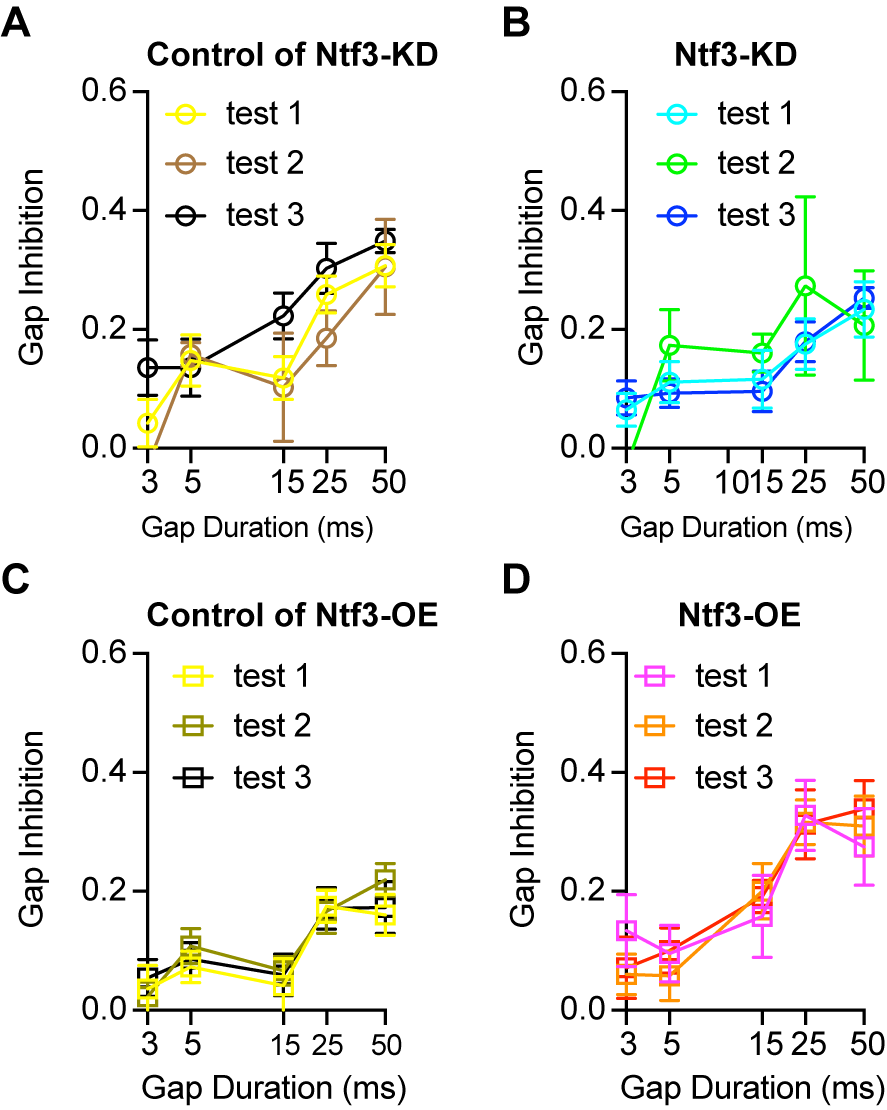

Supplement: S1 Fig — The relationship between gap inhibition vs. gap length does not change between the 3 time points for Ntf3-KD and Ntf3-OE mice. Mean ± SEM are shown. Summary data displayed in S1A–S1D Fig can be found in S1 Data. (TIF) [file pbio.3002665.s001.tif]

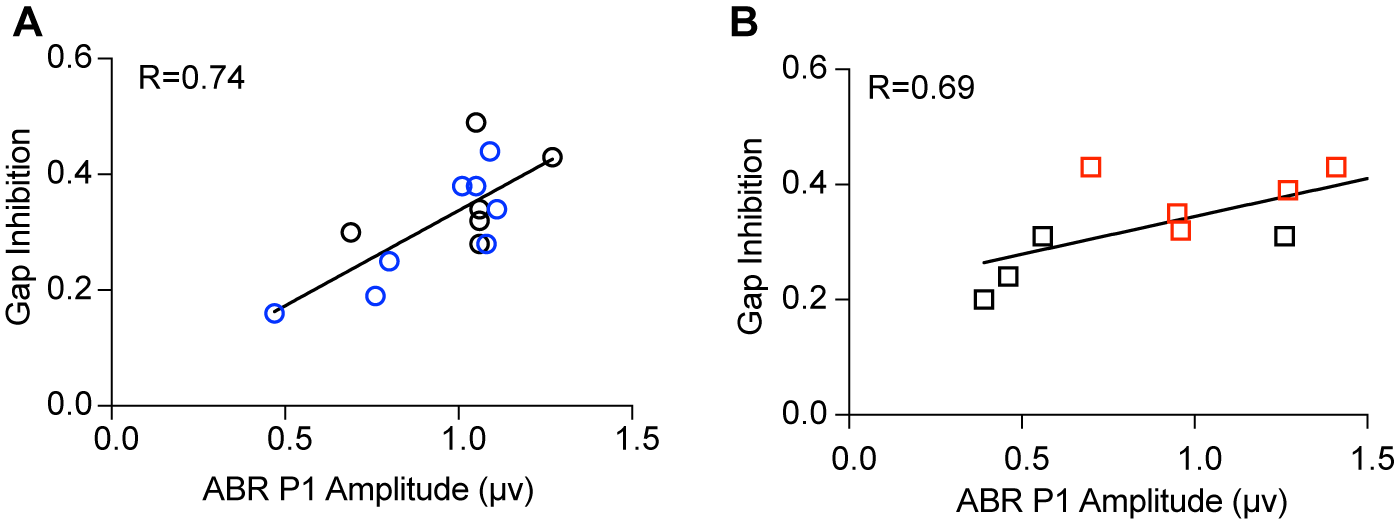

Supplement: S2 Fig — The amplitude of ABR peak I versus gap inhibitory level of Ntf3-KD and their littermate controls (A) or Nft3-OE and their littermate controls (B) show a linear correlation. Summary data displayed in S2A and S2B Fig can be found in S1 Data. (TIF) [file pbio.3002665.s002.tif]
