# Supplementary material for: From hidden hearing loss to supranormal auditory processing by neurotrophin 3-mediated modulation of inner hair cell synapse density
Source: PLoS Biol. 2024 Jun 27;22(6):e3002665. doi: 10.1371/journal.pbio.3002665 (PMC11210788; doi:10.1371/journal.pbio.3002665)
Supplement: Attachment — Submitted filename: Response to reviews 013024.docx [file pbio.3002665.s004.docx]

We thank Reviewer 1 for finding that the manuscript presents interesting results and that we discussed the literature adequately. Here we respond to the important points raised.

Rev. 1: Previous psychoacoustic experiments indicate that good high frequency hearing supports sensitivity to short gaps in noise. Gap detection thresholds are increased (worsened) with high frequency sensorineural hearing loss or with lowpass noise when measured behaviorally using traditional psychoacoustic methods. Though this previous work is not thoroughly addressed in the manuscript, it follows that an increased number of ribbon synapses in the higher frequency cochlear regions could improve processing of gaps as reported in this study. It is not surprising that PPI and GPIAS startle inhibition patterns were different given that typical tone/noise-burst PPI utilizes different circuits than GPIAS, as the authors acknowledge. However, there is some additional data analysis of startle responses in broadband noise that should be performed to support the central conclusion. Additionally, the variability in gap detection thresholds--even in the control groups-should be addressed, as typical gap detection thresholds for mice are on the order of ~2ms, not the >10ms reported here for about 2/3 of the mice. Clarification of these issues is essential to verifying the central claim that "IHC synapse density influences auditory processing but not the startle reflex or sensory gating." There are also some methodological clarifications that would strengthen the manuscript.

Response: we have added references to studies that have shown that gap detection thresholds increase with high frequency sensorineural hearing. The other points are addressed in questions 4 and 9 below.

Methods:
1. The calibration procedures for the various hearing tests are not described. Ribbon synapse counts were determined by a blind observer. ABR and DPOAE thresholds should similarly be determined by a blind observer. Additionally, the authors should describe the threshold determination method. Please provide additional information about how the ABR analysis software identified peaks. Was this analysis supervised by an experienced observer?

Response: We now include this information in the Materials and methods section.

2. How many startle trials +/- 2SD and large PPI or gap startle ratios were eliminated (proportion)?

Response: In the methods section we now explain the criteria by which some (8%-13%) PPI or gap trials were eliminated due to extremely low or high response.

3. Results:
We cannot assume that ABR and DPOAE thresholds remained normal in control or KD/OE groups over the 8 week time period between ABR/DPOAE testing and the final gap detection tests. Hearing status, particularly high frequency hearing, may have been affected over this time with repeated exposure to loud startle-eliciting stimuli and background noise during testing.

Response: It is true that we did not measure hearing thresholds at the end of the experiments. However, it is unlikely that the exposures during the behavioral tests had a significant impact since 65 dB noise is certainly atraumatic and so are 20 msec noise bursts presented at the rate and intensity used in our protocol. Furthermore, gap inhibition for all genotypes was stable across different sessions, suggesting that there were no changes during the 8 weeks of the experiment.

4. Since startle-eliciting stimulus salience is affected by the presence of background noise, the authors should show the ASR amplitudes for the no-gap (broadband noise only) conditions for all groups to confirm that baseline ASR is not different under these conditions. If there is a group difference, then the relationship between ASR in noise (no gap) and gap-induced inhibition should be examined, because there is evidence in mice that prepulse inhibition is related to the strength of the ASR. Increased ASR in noise indicates that the startle-eliciting stimulus is more salient, and this could affect the amount of inhibition that is possible (for example, Csomor et al. 2008).

Response: The analysis has been performed confirming that there was no difference in the baseline ASRs between the groups. See Figure 8B and C.

5. A related issue is that startle-eliciting stimuli presented at saturation for an individual mouse does not produce optimal inhibition (see Longenecker and Galazyuk). A 120 dB startle-eliciting stimulus is almost certainly at the point of response saturation.

Response: The reviewer is correct that our protocol might be almost at the point of saturation. However, that means that, in the worst-case scenario, we have underestimated the performance differences in the different experimental groups.

6. What is the rationale behind choosing 50% ASR inhibition as the gap detection threshold? This is not the 'conventional' method as stated in the manuscript. Previous research has employed several different methods to calculate gap detection thresholds (such as Rd', minimum gap that produces statistically significant inhibition, etc). More sensitive thresholds might be obtained using a different threshold estimation procedure.

Response: Thanks for raising this point, we removed the word “conventionally”. The rationale of choosing 50% of inhibition is that it accounts for individual differences that might occur in maximum levels of inhibition [1, 2], and many studies have used this parameter, e.g. [1-6]. Nevertheless, as suggested by the reviewer, we added Rd’ values (Figure 8H and I) using the method reported by Ison et al., 2005 [7].

7. Both control groups showed extremely variable gap detection thresholds. About half of the Ntf3-KD mice showed gap detection thresholds that were similar to controls, and gap detection thresholds varied immensely in this group from ~2-50 ms. The reasons for this performance variability should be addressed.

Response: We agree that the values we obtained for gap detection thresholds are variable. Yet, our results with the controls are very similar to those reported by others (see figure 4C in Moore et al Nat Comm 2020 [6]).

8. For the Ntf3-OE comparison, the control groups' gap detection thresholds (Fig 8E) varied more than the Ntf3-OE mice. Why were some animals able to perform well while others were not?

Response: The variable efficiency of Cre-mediated gene KO is likely to have contributed to the larger variability in the Ntf3-KD mice.

9. Gap detection thresholds for 'normal-hearing' mice (and most other species tested) are typically ~2ms when measured using GPIAS, traditional operant psychoacoustic methods, and physiological recordings in the inferior colliculus and auditory cortex (e.g. Walton et al.,1997, Ison et al. 2005; Radziwon et al., 2009; Weible et al. 2014). A comparison of the present results to previous gap detection measurements in mice should at least be addressed in the Discussion. One possible explanation for the discrepancy is that the acoustics of the experimental setup were such that the noise offset/offset received by the mouse was not sharp due to echos or restrainer issues, and this may have varied depending on where the individual mouse's head was located (see Ison et al. 2002; Longenecker and Galazyuk 2012).

Response: We added text in the discussion section to discuss different gap detection thresholds reported in the literature and the factors that might contribute to the variability.

10. Additionally, gap detection thresholds measured using startle modification procedures can improve over time due to learning (e.g. Fitch et al. 2008). The authors should compare the GPIAS results over the three test timepoints to determine if there are differences in learning that cause improvements (or potentially habituation) to the gaps.

Response: We compared the GPIAS results over the three test timepoints and found no significant differences, so the GPIAS is constant over time. This is consistent with the findings that gap detection performance in control mice is stable after 60 days [8]. The results can be found in Fig. S1.

Minor Issues:
11. 426: freeze-thawing in 30% of what solution? There seems to be a missing word.

Response: Thanks – it was sucrose – corrected.

12. Line 489: Unclear why reference # 73 is cited here. No GPIAS or PPI experiments were reported in that paper.

Response: That reference was indeed incorrect and has been removed

Rev. 2: Neil J. Ingham – note that this reviewer has signed his review

Rev. 2: We thank Dr. Ingham for appreciating the novelty and significance of our findings. Here we respond to the specific points raised:

1. Ln 121-133. The description of figure 2 and labelling of panels appears to be different in the main text and in the figure legend. In the text, the VGF-Ntf3 correlation is referred to as Fig 2C, but in the legend and on the figure itself, this plot is Fig 2E. The authors should check the figure layout and correct the main text and/or legend accordingly.

Response: Thanks for pointing this out. We have revised the figure accordingly.

2. Ln 144-158 & Figure 3. The pattern of synaptic component counts and density vs cochear region in control mice appears somewhat different - especially in the more basal regions - between the 2 mouse lines. The control curves (in black) are more symmetrical in the Nft3-OE control mice and less symmetrical in the Nft3-KD control mice. Would the authors like to comment on these differences? Is it perhaps related to the different genetic background strain of the 2 lines used? C57BL/6 vs FVB/N?

Response: Yes, the difference in synapse counts between Ntf3-OE and Ntf3-KD mice is most likely caused by the difference in genetic background between strains. These differences were already reported in our Wan et al 2014 elife paper.

3. Ln 168-179 & Figure 4. The estimated ABR & DPOAE thresholds were performed when the mice were 8 weeks old. The subsequent GPIAS testing (involving some high frequency-centred noise bands) was performed up until the animals were 15 weeks old. Given that the C57BL/6 background strain is susceptible to age-related high frequency hearing loss (starting to emerge before 15 weeks old, due to the inherent Cdh23ahl allele), and that the FVB/N are known as "good hearing" mice (according to Jax labs, at least), do the authors feel that such effects may be influencing their behavioural data in any way? A comment relating to this, either in the results or discussion, would be appropriate.

Response: The reviewer raised an important point. We now state that we genotyped all mice to ensure they all carried the wildtype Cdh23 allele.

4. Ln 188-194, Figure 5 & 6. The authors will be aware that it is not only reduced synapse numbers that can results in a reduction of ABR wave 1 amplitude. Other pathologies can also show such effects. An acknowledgment of this would be appropriate.

Response: Yes, other pathologies, for example myelinopathy, can cause peak I amplitude reductions. We now state that the lack of changes in peak I latencies indicates that the defects most likely reflect synaptopathy.

5. Could the authors please explain why they express ABR amplitudes as a normalised value, relative to the control maxima, but that ABR latencies are expressed in absolute millisecond values?

Response: We had used normalized data because the over-expressing and knock-down lines differ in some parameters because they are in different genetic backgrounds. We have reverted the figures to absolute values, since this is what we did in our original description of these lines (Wan et al, eLife 2014)

6. Figure 7. On panels B1 and C1, the schematics of the stimuli, the formatting of the startle stimulus text could be improved, for clarity, to keep it away from the edge of the back box. Perhaps rotate the text 90deg?

Response: Thanks for the comment. We the revised Figure 7 accordingly.

7. Figure 8. Panel A - see comment relating the Figure 7, the same applies here.

Response: Thanks for the comment. We the revised Figure 8 accordingly.

8. Figure 8. Panels B & C. What type of line / curvefit is used here? Panel D. It appears that approximately half of the gap detection thresholds from the Nft-KD mice are comparable to control values. Was there any correlation between these mice / points with any other parameters measured in the study, eg. ABR wave 1 amplitude? For example, did the mice with more normal-looking gap detection thresholds have ABR amplitudes / synaptic density more similar to controls, or those with poor gap-detection thresholds have relatively very small ABR amplitudes / synaptic density?

Response: As detailed now in the methods section, to calculate the gap-detection threshold, the data were fitted with a three-parameter logistic function. In Fig. S2 we now also show that there is a strong correlation between GPIAS inhibitory levels and ABR P1 amplitudes for both lines.

Rev. 3: Anthony Ricci - note that this reviewer has signed his review.

We thank Dr. Ricci for his positive comments on the paper. Here we respond to his specific points regarding “presentation clarity for a broad audience as well as for interpretation of data”.

1- The data does suggest that synaptopathy as defined here causes temporal processing deficit, but it does not directly suggest this same mechanism is a key contributor to HHL. This seems like a bigger leap than is supported by the data, at least for the abstract.

Response: thanks for the comment – we changed the abstract to clarify this point.

2- It seems like a control for central effects would be to treat with tamoxifen after synaptic pruning was completed within the cochlea. This would leave the central effects intact but potentially remove the peripheral changes. I am curious as to why this was not done?

Response: Dr. Ricci is correct and what he suggests would be an interesting follow-up study. The current experimental design was an important first step. We already demonstrated that induction of Ntf3 over-expression in young adult mice does not alter ABR peak 1 amplitudes and synapse densities (see Cassinotti et al, Aging Cell). Also, in the Aging Cell paper we showed that Ntf3 over-expression in middle-aged mice rapidly increases ABR peak 1 amplitudes without changing thresholds – it will be interesting to see if there is also a rapid Ntf3-induced improvement in gap detection at that age. Those studies are in planning stage and will take a long time to generate the mice.

3- I think it is important to show raw data traces whenever possible. This is particularly true for a broader audience journal where ABRs and DPOAEs are not standard. I suggest adding these examples for each condition tested. In addition, a more detailed description and example of how the various peaks were measured independently of each other is also important.

Response: As suggested, representative traces of DPOAEs and ABRs and information on the frequencies and stimuli intensities have been added to Figure 4 (panel G and H). we also provide more detail explanation of threshold and peak identification.

4- I don't see the value in normalizing the data, particularly when it is across animal populations. In figure 3 simply showing the absolute counts would be more meaningful. The compressed y-axis doesn't really help with assessing the extent of the changes. Similarly normalizing amplitudes or each wave again seems an unnecessary manipulation as compared to showing absolute values.

Response: We had used normalized data because the over-expressing and knock-down lines differ in some parameters because they are in different genetic backgrounds. We have reverted the figures to absolute values, since this is what we did in our original description of these lines (Wan et al, eLife 2014)

5- Given that mice operate at frequencies higher than the 32kHz tested, shouldn't higher frequencies be assessed?

Response: Actually, we show data at 45 kHz (Fig 4) and up to 42 kHz for GPIAS (Fig 9).

6- Was there any correlation between the gap detection thresholds and the synapse number changes for the Ntf3-KD?

Response: Unfortunately we didn’t test gap detection thresholds on the mice used for synapse counting.

We only have data for gap inhibition and ABR P1 amplitude from the same mice.

7- Do the authors know if a particular fiber type is preferentially impacted by the treatment or if the relative fiber type distribution is altered by the reduced pruning?

Response: No

8- Is there evidence for increased calcium currents or number of calcium channels due to the increased synapse numbers?

Response: No

References

1.	Ison, J.R. and G.P. Bowen, *Scopolamine reduces sensitivity to auditory gaps in the rat, suggesting a cholinergic contribution to temporal acuity.* Hearing Research, 2000. **145**(1): p. 169-176.

2.	Barsz, K., et al., *Behavioral and neural measures of auditory temporal acuity in aging humans and mice.* Neurobiology of Aging, 2002. **23**(4): p. 565-578.

3.	Ison, J.R. and L.A. Pinckney, *Reflex inhibition in humans: sensitivity to brief silent periods in white noise.* Percept Psychophys, 1983. **34**(1): p. 84-8.

4.	Walton, J.P., et al., *Neural correlates of behavioral gap detection in the inferior colliculus of the young CBA mouse.* J Comp Physiol A, 1997. **181**(2): p. 161-76.

5.	Ison, J.R., et al., *The relative detectability for mice of gaps having different ramp durations at their onset and offset boundaries.* J Acoust Soc Am, 2002. **112**(2): p. 740-7.

6.	Moore, S., et al., *A role of oligodendrocytes in information processing.* Nat Commun, 2020. **11**(1): p. 5497.

7.	Ison, J.R., et al., *The behavioral response of mice to gaps in noise depends on its spectral components and its bandwidth.* J Acoust Soc Am, 2005. **117**(6): p. 3944-51.

8.	Kaylegian, K., et al., *5XFAD Mice Show Early Onset Gap Detection Deficits.* Frontiers in Aging Neuroscience, 2019. **11**.
